# Recent Views of Enteric Fever

**Published:** 1890-06-28

**Authors:** 


### The Practitioner's Mirror and Retrospect.

*[The Editor will be glad to receive offers of co-operation and contributions from members of the profession. There is no doubt that much valuable material full of interest and instruction is at present lost to science. This is largely so in the case of* (1) *the younger and less-known members of the hospital staff* (2) *those connected with cottage hospitals, and* (3) *the great body of medical practitioners not attached to any institution. The co-operation of all is cordially invited to enable the Editor to make* The Hospital *of the utmost possible use and value to the profession. Specialists willing to review books on their special subjects are invited to communicate this fact at once. All letters should be addressed to* The Editor, The Lodge, Porchester Square, London, W.]

### MEDICINE.

### RECENT VIEWS OF ENTERIC FEVER.

The characteristics and symptoms of typhoid or enteric fever, as given in text books, are sufficiently distinctive. It may be defined as a continued fever attended with diarrhœa, and characterized by eruption on the skin, and intestinal lesion. But it often appears under very diverse forms, sometimes one symptom being the prominent feature, and at other times another being most manifest. In this respect it is not peculiar, many other diseases showing variation of symptoms from the type, although few do so to the extent of typhoid fever. One of the most recent authors (Taylor) says, "Few diseases are more variable than typhoid fever." And it is well that this should be recognized more distinctly than was the case in the older text-books.

Dr. T. W. E. Hare, of Brisbane, writing quite recently in an Australian medical journal, states that the type of typhoid fever in that locality has recently changed, and that a milder form of typhoid has been persistent since the commencement of 1887. But this change of type, while it lowered the mortality from pyrexial causes, left the dangers arising from intestinal lesion unaffected. As is well recognized, perforation of the bowels is one of the great dangers in typhoid fever. Quite recently the report of a case of the kind "went the round of the press," perforation having been caused by the imprudence of relatives who brought a hospital patient improper things to eat. Perforation of the bowels is most likely to occur between the twenty-fifth and thirty-second days of the disease, an accident usually attended with symptoms of collapse, and according to most text-books always proving fatal. Hence the care which physicians take that their enteric fever patients should have no solid food under six weeks or two months. Eating an orange, or a piece of potato, or drinking an effervescent draught may, it is believed, cause distension and rupture of the healing bowels. It does not appear, however, that perforation is always immediately fatal. A case was recorded in the *Lancet* some time ago of perforation not proving fatal for fifty days, the post mortem showing adhesion between two layers of the ileum. Neither does it seem that perforation is necessarily fatal.

Mr. G. A. Branson recently detailed a case of "typhoid fever perforation and recovery." The patient was a professional nurse, who ran through an ordinary attack of typhoid fever until the twentieth day, when she sat up in bed, and took a cupful of broth. This was followed by a relapse. From the symptoms detailed, Mr. Branson thinks the fact of perforation appears established. On the thirty-first day she complained of feeling very sick, with considerable pain in the right cæcal region, respiration short and hurried, pulse 130, temperature 130 deg. A few hours afterward the pain in the abdomen was almost gone, the skin cold, with clammy sweat, pulse imperceptible, violent retching, abdomen commencing to swell, and hiccough. On the thirty-second day the temperature had fallen to 98 deg., the abdomen was intensely distended, and the pulse imperceptible. She continued in this state for several days, but on the thirty-sixth day began to improve. On the thirty-eighth day she passed a number of small sloughs, and on the fortieth day was convalescent.

In a very recent article by Dr. T. F. Maclagan, physician to their Royal Highnesses Prince and Princess Christian, on the abdominal distension of typhoid fever, Dr. Maclagan observes that a moderate amount of tympanitis is a common symptom not calling for special treatment. But the distension may be so great that by upward pressure it embarrasses the breathing, causing much distress, and oftentimes danger, especially if it comes on late when the patient is much reduced. Sloughs and sloughy discharge have been slipping through the ileocæcal valve into the cæcum, and if not got rid of by stool, gas forms from decomposition, and the bowel becomes paralyzed from over-distension. When such a point is reached, the patient is in danger, and this distension may be developed rapidly. Dr. Maclagan says there are two remedies, one by tapping the colon with a fine trocar, the other by passing a long tube *viâ* the rectum. The latter plan he regards as less risky and most efficacious, and cases are recorded of the timely introduction of the tube apparently saving life.

In the report of the Surgeon-General, United States Army, for the year ending June, 1889, it is remarked that "as abortive cases of typhoid fever are often apocryphal, or at least rare," a case is given. There was no evident fever, and little appetite, but the patient ate the regular ration, which gave rise to much intestinal irritation, causing a rise of temperature to 106 deg. F. But after purgation there was rapid recovery. We do not, however, feel by any means assured that this was a case of typhoid fever, and it appears to have been considered as such principally from the fact of typhoid fever prevailing in the locality. But that there are abortive cases of typhoid does not admit of question. Dr. A. McPhedan, Lecturer on Clinical Medicine at the University of Toronto, has observations in the last *Canada Lancet* on abortive forms of typhoid fever. As with scarlet fever, diphtheria, and most other maladies, there are very severe and very mild cases. "There are, indeed, few, if any, diseases which do not in some instances run an abortive course." But it is only recently that we meet with a notice of abortive typhoid fever cases in standard works. In former editions of "Flint," "Bristowe," "Reynolds' System," etc., no reference is made to it. The author believes that many attacks of malaria, headache, feverishness, and perhaps diarrhœa, which are usually attributed in Toronto to malaria, are really typhoid. Now, we are fully of opinion that malaria as a cause of disease has been too greatly credited, and we agree with Dr. McPhedan, that various conditions which have been attributed to malaria are rather typhoid in origin. However this may be, there is no manner of doubt that the intestinal lesions of typhoid may progress without any of the constitutional symptoms being present. A case of latent typhoid fever was recorded some time ago in the *British Medical Journal*. The patient never complained of any symptoms, but died rather suddenly from cardiac thrombosis. At the autopsy it was found that Peyers patches exhibited every stage of enteric fever. Another case was reported in the *Lancet* in 1887 of latent typhoid not diagnosed during life, death occurring from perforation of the ileum and sudden collapse. Very recently Dr. William Graves, of St. Louis, Mo., published a report of a case of typhoid fever, with an unusual complication. A girl had all the symptoms of typhoid after attending her mother through the disease, but the rash which appeared at the usual time was distinctly vesicular, and in several places pustular. It is known that a "scarletiform" rash sometimes precedes the rose eruption of typhoid, and scarlatina has been known to be associated with typhoid. But in this instance scarlet fever was out of the question, as the girl had previously had this affection, and the rash was not a scarlet fever one. Variola was also excluded for precisely similar reasons. Dr. Graves concludes that this was a case of "atavistic" typhoid. "May we not assume," he asks, "that the eruption as observed was the original eruption of typhoid, and that during the course of time it has become so modified that we now only have rose spots?"

A recent author gives the varieties of typhoid fever as below. First, there are instances of what has been called "walking cases of enteric fever" or typhoid ambulous, when the patient, although suffering from the principal characteristics of the malady, does so in so mild a form that he does not require to interrupt his ordinary avocations. Secondly, there is mild typhoid, when the person, although confined to the house or bed, is never in a dangerous condition. Thirdly, there is abortive typhoid, when the malady terminates suddenly about the seventh to the twelfth day. Fourthly, there is intermittent typhoid, when cases of enteric fever present intermittent characteristics from first to last. Fifthly, there is the remittent form of typhoid, and in this form there is often bilious purging and vomiting, so that it has been also styled "bilious typhoid." Sixthly, enteric fever may set in suddenly with rigors, instead of the usual period of malaise. Seventhly, symptoms referable to the stomach may predominate throughout, mastering every other symptom; hence one of the terms applied to the disease, viz., "gastric fever." It is this difference of symptoms which has led to typhoid fever being described under so many different names. And it is this difference in the symptoms which caused the differentiation of typhoid and typhus to be of comparatively recent date. Now experts desire us to believe that typhoid fever is to be diagnosed by the discovery of the bacilli, which are supposed to be characteristic. But the discovery of the bacilli is oftentimes a difficult matter.

Dr. H. Neumann, of Berlin, has recently turned his attention to the discovery of typhoid bacilli in the urine, for the importance of the discovery of the bacilli as a diagnostic measure had led physicians to puncture the spleen in search for them. One hundred and fourteen examinations of the urine in forty-eight cases were made, and bacilli met with in eleven cases. It is stated that when the bacilli were in numbers their presence might be suspected from the turbidity of the fluid. The identity of these bacilli with typhoid bacilli was proved by isolation and pure cultivation. As, however, colonies of bacilli are not formed in the kidneys before they are in the skin, they cannot be expected to appear in the urine before the rash on the body. It is, however, desirable that we should be able to make a diagnosis at an earlier date. We recollect an author stating the diagnosis between remittent, as it occurs in the East, and typhoid could often only be accomplished by observing the effect of quinine, which would be beneficial in the former and useless in the latter. At present, early diagnosis depends pretty much on the prevalence or otherwise of enteric fever in the neighbourhood!

We observe an article in the *New York Medical Record* for March 8th, by Dr. R. F. Licorish, on the value of absolute rest and starvation in the early stages of typhoid fever. He instructs the nurse not to give any nourishment until the patient calls for it. The result of the treatment is said to be soon apparent, for after a few days desire for food returns. By starvation the stomach gets rest, all effete material is carried off, the catarrhal condition is reduced, and the temperature shows decline. By this means Dr. Licorish thinks the fever is aborted, and that the cases treated as above were typhoid he considers proved by relapses occurring in some, which being otherwise treated, the patient passed through the ordinary stages of the malady. After a normal temperature has been gained the patient should not be allowed to sit up for at least ten days.

Mr. F. W. Elsner, of Melbourne, has recently pointed out, that by supplying a typhoid fever patient with beef tea, chicken broth, and the like, he is given the very materials used in bacteriology for cultivations, in which micrococci develop best and increase prodigiously. It is now rarely that his typhoid fever patients get beef tea or other bacteriological breeding fluid as their diet. In the Hospital "Retrospect" for January 25 we observed, there is an objection to beef tea, as it may contain peptones. The older physicians held that the free administration of food in fever occasionally intensifies the febrile process, and we feel quite sure that the indiscriminate administration of concentrated foods in febrile diseases is often distinctly injurious. We believe that milk, if it can be obtained good and without being too much adulterated, is the best food for fever patients. But it requires to be diluted with pure water, or with Apollinaris water. And we desire to make this dilution ourselves; not having it made for us by a provider in the country, who recently being summoned for adulterating milk confessed to mixing it with water, to what exact extent he did not know! With reference to the distribution of enteric fever, we observe that Dr. Robert Grieve, Surgeon-General, British Guiana, in a recent address to the Guiana Branch of the British Medical Association, remarks there was no record of a clear and unmistakable case of enteric fever of local origin. He thinks that typhoid is one of the diseases in regard to which the races of men vary much in their susceptibility. He also states that it is pre-eminently a disease of white races. But against this conclusion there is the fact that the natives of India suffer from it. But with regard to the symptoms, Dr. Grieve says he is not inclined to accept any one symptom separately as constituting the disease, nor is he inclined to accept a single pathological fact as constituting the disease. With this we are quite in accord. For ulceration of Peyers patches has been found when typhoid fever did not exist. In connexion with the above, it may be mentioned that several reports of typhoid fever occurring in phthisical patients have recently appeared. It is known that tuberculosis may occasion very similar appearances as the intestinal lesions of enteric fever. Such lesions have also been noticed in connexion with various other ailments. Indeed, it would appear that, as observed by Dr. Harley, enteric fever and its attendant phenomena may occasionally become a part of almost any other morbid condition.

